# Comparing Objective Measures of Sleep Disturbance and Sleep Related Impairments as Proximal Risk Indicators of Suicidal Intent and Non‐Suicidal Self‐Injury

**DOI:** 10.1111/sltb.70091

**Published:** 2026-03-31

**Authors:** Nicolas Oakey‐Frost, Emma H. Moscardini, Megan L. Rogers, Jennifer J. Muehlenkamp

**Affiliations:** ^1^ VA Salt Lake City Healthcare System Salt Lake City Utah USA; ^2^ McLean Hospital, Harvard Medical School Belmont Massachusetts USA; ^3^ Department of Psychology Texas State University San Marcos Texas USA; ^4^ Department of Psychology University of Wisconsin Eau Claire Wisconsin USA

**Keywords:** actigraphy, daily diary, NSSI, passive sensing, sleep quality, suicidal ideation

## Abstract

**Background:**

Non‐suicidal self‐injury (NSSI) and suicidal ideation (SI) are prevalent public health concerns among college students. Poor sleep quality and its consequences are a risk factor for NSSI and SI in this population. Unfortunately, limited research measures the impact of sleep related impairment (SRI) on NSSI/SI, little research *compares* the relative criterion validity of objective versus SRI measurement for NSSI/SI, and there is limited research measuring NSSI *behaviors* as a primary outcome.

**Methods:**

This study examined the relationship between passively captured Consensus Sleep Diary indices (i.e., actigraphy), SRI, SI, and frequency of NSSI in a sample of *N* = 132 college‐students reporting past‐month NSSI/SI. Participants wore actigraphy wristbands during the 28‐day daily ambulatory phase and completed self‐report assessments each day (*k* = 3726 observations).

**Results:**

Multilevel mixed effects models failed to find significant relationships between actigraphy‐derived sleep quality indices, same‐day SI and NSSI frequency, and next‐day SI and NSSI frequency. Significant relationships between SRI, same‐day SI, and same‐day NSSI frequency emerged.

**Discussion:**

Results suggest worsening SRI may be an important signal of worsening SI and NSSI frequency. Implications for assessment and monitoring of SRI for college students at risk for NSSI and suicide are discussed.

## 1 Introdcution

College students are at particularly high risk for suicide due to sleep disturbances, which are widely considered significant precipitants of suicidal crises (Fernández Rodríguez and Huertas 2013; Garnett and Curtin 2024; Russell et al. 2019). Over 50% of college‐students report getting only 5–7 h of sleep every night, and over 62% are classified as “poor sleepers” (Becker et al. 2018). Such significant sleep disturbances among college students are concerning given evidence of consistent and significant longitudinal relationships between sleep disturbances and suicide thoughts and behavior (STB; Liu et al. 2020). In fact, the pooled effect sizes linking sleep problems and suicide ideation (SI) are markedly larger for shorter follow‐up periods; as such, daily diary and ecological momentary assessment (EMA) can tease apart the intersecting dynamics of these phenomena (Liu et al. 2020).

Ambulatory methods like EMA and daily diary allow scientists and clinicians to gain invaluable insights into the dynamics of risk and protective factors driving the emergence of suicidal crises of the short‐term (Czyz et al. 2021; Kleiman et al. 2017). Prevailing scientific and clinical experience suggests that acute sleep disturbances are a warning sign for an impending suicidal crisis; however, the available evidence appears modest. In fact, Liu et al. (2020) identified only a few studies examining the short‐term, longitudinal relationships between sleep disturbances and STB, and most of this work relied on subjective reports of sleep (Bozzay et al. 2025; Rogers and Bozzay 2024). More ambulatory research is required to substantiate the relationship between acute sleep disturbances and worsening STB over short periods.

Integrating passive wearable technology with ambulatory design is a promising avenue for assessing relationships between sleep disturbances and STB. Actigraphy wearables calculate various sleep quality indices based on physiological movement through accelerometry (Bernert et al. 2019; Kivelä et al. 2023; Kearns et al. 2023). Typically, actigraphy‐based measurements coincide with the Consensus Sleep Diary (CSD), measuring total sleep time (TST), sleep onset latency (SOL), sleep efficiency (SE) and wake after sleep onset (WASO). Through the integration of passive wearables with daily diary questions targeting sleep health, scientists and clinicians may gain additional insight into the specific indicators of sleep disturbances driving STB over time.

One area of the sleep disturbance and suicide research nexus requiring further examination is the relative criterion validity of actigraphy derived sleep quality indices versus day‐to‐day changes in sleep‐related impairments (SRI), defined as self‐reported perceptions of alertness, sleepiness, tiredness, and functional impairments during wakeful hours that arise due to sleep disturbances (Patient‐Reported Outcomes Measurement Information System [PROMIS] 2020). SRIs may be important clinical indicators of STB risk given recent studies demonstrating that subjective appraisals of sleep quality and sleep‐related cognitive or affective impairments offer stronger incremental validity for STB than objectively appraised poor sleep quality (Glenn et al. 2021; Hamilton et al. 2023; Mournet et al. 2025). Self‐reported improvements in SRI, but not sleep‐related disturbance, have also been shown to explain the relationship between treatment for sleep‐related disorders and improvements in self‐rated functioning among those with serious mental illness (Armstrong et al. 2022). These results suggest SRIs may play an important role in recovery from symptoms of serious mental illness and possibly STBs. Measuring changes in SRI in tandem with actigraphy and daily STB may provide actionable insights toward understanding and preventing suicide.

## 2 Actigraphy, Sleep‐Related Impairments, and Suicidal Ideation

A few studies have examined the relationship between actigraphy derived sleep quality indices and SI with mixed results. Bernert et al. (2019) observed that only sleep variability (i.e., standard deviations of sleep onsets and offsets) predicted worsening SI over a three time‐point 21‐day study of university undergraduates; the self‐report sleep diary variables were not significantly prospectively associated with worsening SI at follow‐up. More recently, *subjectively reported* CSD indices demonstrated a significant relationship with same‐ and next‐day SI in daily diary or EMA methodological investigations; however, *actigraphy derived indices* of sleep quality were observed to be unrelated to next‐day SI (Glenn et al. 2021; Kivelä et al. 2023). Concordant with observations and results of Czyz et al. (2023) passive sensing study, using objectively or passively assessed sleep quality indices to predict changes in SI are mixed at best, highlighting a need for further study and scrutiny.

Unfortunately, scientific work testing short‐term relationships between SRI and STB using ambulatory methodologies is scant. However, Nadorff et al. (2014) observed that changes in sleep‐related daytime fatigue at admission to inpatient psychiatry significantly predicted worsening SI at discharge independent of effects of self‐reported depression and SI at admission. Loss of energy and fatigue due to poor sleep were also significantly related to worsening SI at discharge compared to patients whose sleep improved during inpatient treatment. Relatedly, Hamilton et al. (2023) observed that actigraphy‐measured sleep changes were unrelated to SI over time; however, individual decreases in sleep time were significantly related to worsening SI indirectly through higher affective reactivity. A case series analysis of seven adults with borderline personality disorder undergoing treatment observed that sleep‐related daytime fatigue was associated with SI, NSSI urges, and desire to be alive in three of the seven patients within the case series (Mournet et al. 2025). Taken together, some evidence suggests self‐reported perceptions of SRIs during wakeful hours are associated with changes in SI over time, but additional research is required.

### 2.1 Actigraphy, Sleep‐Related Impairments, and Non‐Suicidal Self‐Injury (NSSI)

Unfortunately, the relationships between poor sleep quality and self‐harm *behavior* like non‐suicidal self‐injury (NSSI) are not as well‐studied (Tubbs et al. 2022). Nearly 20% of first‐year college students report engaging in NSSI during their lifetime and 8% report engaging in NSSI in the past year (Kiekens et al. 2023). Population‐based cohort studies have demonstrated a significant link between lower TST and NSSI history among adolescents while greater discrepancies between weekday and weekend sleep schedules are associated with a 17% greater risk of recent NSSI occurrence (Hysing et al. 2015; Tubbs et al. 2023). Asarnow et al. (2020) observed that a one standard deviation increase in self‐reported sleep problems may predict a nearly two‐fold increase in odds of NSSI frequency within 1 month. The aforementioned evidence signals a potentially strong relationship between sleep‐related disturbances and NSSI; however, EMA and daily diary studies leveraging actigraphy or self‐reported SRI have rarely been used to understand the relationship between sleep quality indices and NSSI behavior.

A recent study from Burke et al. (2022) used a 10‐day ambulatory assessment protocol to examine the relationship between actigraphy derived objective measures of sleep quality and *NSSI intent*. Changes in a composite variable (i.e., changes in napping, night wakening, sleep timing, and sleep duration) were associated with greater *NSSI intent* severity each day over the course of the 10‐day ambulatory phase (Burke et al. 2022). While promising, the results fail to distinguish between independent objective indicators of sleep quality (e.g., SOL and WASO) within models and fail to test the relationship between these objective measures of sleep quality and NSSI *behavior*. As Tubbs and colleagues (2022) argue, using composite indices, especially within actigraphy, fails to account for granularity in distinct sleep related processes and may even increase risk of multicollinearity within statistical analyses.

### 2.2 This Study

Poor sleep quality, SI, and NSSI are highly prevalent comorbidities among college students in the USA, and only a few available ambulatory studies have attempted to link daily changes in sleep patterns to SI and NSSI. In fact, very few studies have examined and compared the relative utility of actigraphy derived CSD with measures of SRIs as predictors of change in STB over days and weeks using ambulatory methods in high‐risk samples. This is despite growing evidence that cognitive and affective impairments due to poor sleep quality may be stronger predictors of STB than objectively assessed sleep quality indices (Glenn et al. 2021; Hamilton et al. 2023; Mournet et al. 2025; Nadorff et al. 2014; Littlewood et al. 2019). Comparing the effects of SRI appraisal with objectively derived sleep quality indices could provide important, fine‐tuned clinical indices and contrasts to characterize the relationship between sleep quality, SI, and NSSI.

This study aimed to examine the impact of objectively assessed sleep quality indices on same‐day and next‐day suicidal intent and NSSI frequency. It was hypothesized that H1 objectively measured indices of sleep quality would be *unrelated* to same‐day and next‐day suicidal intent and H2 NSSI frequency (Glenn et al. 2021; Kivelä et al. 2023; Czyz et al. 2023). Next, this study aimed to examine the impact of subjectively appraised SRI on same‐day and next‐day suicidal intent and NSSI frequency, and if significant models emerged between the two forms of sleep quality appraisal (objective vs. subjective SRI), conduct statistical comparisons of the models to determine which method of assessment is best suited to detecting change in suicidal intent and NSSI frequency. It was hypothesized that H3 subjective appraisals of SRI would be significantly related to and significantly predict same‐day and next‐day suicidal intent and H4 same‐day and next‐day NSSI frequency (Glenn et al. 2021; Kivelä et al. 2023; Czyz et al. 2023).

## 3 Methods

### 3.1 Participants

Participants included 132 young adults (*M*_age_ = 19.44, SD = 1.43) with a past‐month history of NSSI and non‐zero level of suicide ideation. The sample was predominantly cisgender women (69.7%) with 11.4% identifying as nonbinary, 9.1% as cisgender male, 3.8% gender fluid, 3.1% transmasculine, and 2.9% declined to answer. Most of the participants identified as White (93.2%), with 6.1% identifying as Asian/Pacific Islander, 0.7% as Black/African American, and 2.3% reported a Hispanic/Latine ethnicity. Just under half the sample (49.2%) reported currently being in counseling, and 63.6% reported having a prescription to manage a psychiatric disorder.

### 3.2 Procedures

Participants were recruited from an online screening questionnaire sent to a random sample of undergraduate students at a Midwestern U.S. University. Screening participants who reported at least one episode of past‐month NSSI, a non‐zero level of past‐month SI, and provided contact information were invited to participate in the full study. Participants completed informed consent procedures and a subsequent battery of questionnaires on a computer in the research lab. Following the battery, participants were fitted with a Phillips Respironics Actigraph 2 watch and instructed on its use. Participants were asked to wear the watch 24 h/day and to press a side button indicating intentions to sleep and upon waking. The parent study from which the current data were obtained included four additional weekly return visits for assessment. Participant sleep data was downloaded and reviewed for accuracy during weekly return visits to the lab as part of the parent study.

Participants were also instructed on the daily diary phase during the baseline visit. For 28 days, participants completed a 20‐item daily diary survey, emailed nightly via Qualtrics at a time of their choosing between 8 and 11 pm. Responses indicating current suicidal intent (score of 6 or higher on 8‐point scale) and/or low ability to resist the urge to kill oneself (score of 4 or lower on 8‐point scale) were flagged and an automatic alert was sent to the primary investigator who conducted a safety screening check in accordance with best practice guidelines (Nock et al. 2021). Compliance was monitored and participants with less than 70% completion during the week problem‐solved ways to improve adherence with research staff during their weekly return visits to the lab. After each visit to the research lab, participants reviewed their safety plan and were provided with campus, local, and national free mental health resources before leaving. All study procedures were approved by the IRB affiliated with the last author.

### 3.3 Materials

#### 3.3.1 Objective Sleep Quality

Sleep–wake patterns were analyzed continuously using the Phillips Respironics Actigraph 2 Watch which assesses movement and light exposure (sampling rate: 32 Hz; epochs: 15 s), representing a gold standard method for objectively assessing sleep quality. Actigraph software automatically detects major rest intervals by considering the activity count of the current 1‐min epoch against the two preceding minutes and following it to determine sleep/wake status. To ensure quality data, the rest interval was set to high sensitivity (> 3 h) and participants were instructed to push the watch event marker indicating intentions to sleep and upon waking. Participant sleep data were reviewed at weekly lab visits to identify any non‐wear time and manually adjust artifacts and extreme/implausible values prior to software calculation of sleep indicators. The standardized scoring algorithms built into the actigraph software program (Actiware, Philips Respironics, Bend, OR) provided calculation of sleep activity and quality across different sleep variables including: sleep onset latency (SOL; minutes taken to fall asleep), total sleep time (TST; total minutes spent sleeping per 24 h period), wake after sleep onset (WASO; time awake after sleep onset [minutes]), sleep efficiency (SE; % time asleep/time in bed). Each variable was calculated per day across the 28 days of the study.

#### 3.3.2 Sleep‐Related Impairments

Two items from the PROMIS Sleep Related Impairment subscale (Yu et al. 2012) were used to evaluate subjective SRI: “I had a hard time getting things done because I was sleepy”, and “I had problems during the day because of poor sleep*”*. These items were chosen because they demonstrated a strong ability to discriminate between participants with high and low sleep disturbance within psychometric analyses during creation of the short form; they also evaluate general functional impairment versus specific domain impairment due to poor sleep quality (Yu et al. 2012). Only two items were used to reduce participant response burden. Each item used a 5‐point Likert‐type scale ranging from (0) *not at all* to (4) *very much*. These items were highly correlated (*r* = 0.80; see Table 2); thus, they were summed for each participant across time points to create a total, subjective SRI score for each participant, each day.

#### 3.3.3 NSSI Behavior

The frequency of engagement in NSSI was assessed with a single item, “How many different times did you engage in self‐injury today?” Response options ranged from (0) none to (5) five or more times.

#### 3.3.4 Suicidal Intent

A single item based on previously published EMA studies of suicide (Kleiman et al. 2017) was used to measure suicidal intent: *How strong is your intent to kill yourself today?* Response options ranged from (0) Not at all to (7) Extremely Strong.

### 3.4 Analytical Plan

Power analysis was conducted assuming repeated measures design using Optimal Design Software (Raudenbush et al. 2011). To detect a small to medium effect (*d* = 0.35), with alpha = 0.05, power = 0.80, daily assessments across 28 days, and correlations among repeated measures = 0.50, a minimum sample size of approximately 132 participants was required. Descriptive statistics of both the demographic makeup of the study sample and STB were calculated. Two duplicate participant entries and one mislabeled date entry case were removed from the dataset. No missing data were removed from the dataset during formal analyses. Next, repeated measures correlations were run to examine within‐person relationships in study variables over time.

We then examined the relationship between objectively measured indices of sleep quality, SRI, suicidal intent (i.e., SI), and frequency of engaging in NSSI concurrently (i.e., within‐day) and prospectively (i.e., next‐day). Random intercept linear fixed effects models estimated via maximum likelihood (ML) were used to test the effect of objective and the SRI variable on within‐day (*T*) and next‐day (*T* + 1) suicidal intent over the course of the study follow‐up period; random slopes models were examined but did not converge. Cumulative link mixed effects models using Laplace approximation and Hessian optimization were used to test the relationship between the independent variables and same‐ and next‐day NSSI frequency; this method was used to account for the ordinal nature of the NSSI question. All independent variables were within‐person mean centered prior to analysis. Each prospective, “next‐day” model included the past‐day dependent variables as covariates to control for past‐day occurrences of each outcome as probable predictors of next‐day occurrences of SI and NSSI.

The models examined the effect of past‐evening objectively assessed sleep indices and SRI on same‐day suicidal intent and NSSI frequency. Each of the within‐person mean centered objective sleep indices (i.e., TST, SOL, SE, and WASO) predicting same‐day suicidal intent and NSSI frequency were entered into separate regression models to avoid multicollinearity (cf. Littlewood et al. 2019). Repeated measures correlations demonstrated significant bivariate relationships between all actigraphy derived CSD indices which ranged from small to moderate in magnitude (see Table 2). Next, the within‐person mean centered SRI was entered into a single model to test for same‐day relationships between SRI, SI, and NSSI frequency. If any of these models emerged as statistically significant, the a priori intent was to compare the superiority of the model's objective and SRI models using AIC, BIC, and loglikelihood difference tests.

The prospective, “next‐day” models examined the effect of past‐evening objectively assessed sleep indices and SRI on next‐day SI and next‐day NSSI frequency (*T* + 1). For objective indices, within‐person mean centered objective sleep indices and past‐day SI and NSSI frequency were entered into separate models, predicting next‐day SI and NSSI frequency for each objective index. Next, the within‐person mean centered SRI and past‐day SI and NSSI frequency were entered into a single model to test for significant relationships between SRI, SI, and NSSI at *T* + 1. If any of these models emerged as statistically significant, the a priori intent was to compare the superiority of the model's objective and SRI models using AIC, BIC, and loglikelihood difference tests. All analyses were conducted in R, 2024, version 12.1 + 563.

## 4 Results

See Table 1 for a complete participant demographics and daily diary measurement characteristics included in this study (i.e., ICC, RMSSD, and RMSSD range). ICC estimates indicated approximately 49%–82% of variability in item responses is attributable to within‐person, as opposed to between‐person, variance. RMSSD values illustrated significant variability in responses between time points (e.g., over 150 min of change in TST). During the 28‐day daily diary phase, a total of *k* = 3726 observations were collected. Participants completed an average of *M* = 28.66, SD = 2.98 (range = 10–28 possible surveys). Several participants completed two to three surveys beyond the 28 days based on scheduling of final debriefing interviews; one participant with 41 completed surveys was an outlier. These extra surveys were retained during analyses. Across the daily diary phase, there was a cumulative total of 12.2% missing data; missing data for actigraphy were higher, at 18.4%. Item level missingness for study variables can be found in Table S1. No missing data were removed from the dataset during formal analyses. A repeated measures correlation matrix for study variables can be found in Table 2.

**TABLE 1:** Descriptive and demographic data for college student sample (*N* = 132).

Demographics	*M* (SD)	ICC 95% CI [LB; UB]	RMSSD (SD)	RMSSD range
Age	19.44 (1.43)			
Gender				
Cisgender female	69.7%			
Nonbinary	11.4%			
Cisgender male	9.1%			
Gender fluid	3.8%			
Trans‐masculine	3.1%			
Decline	2.9%			
Ethnicity				
Non‐hispanic	97.7%			
Hispanic	2.3%			
Race				
White	93.2%			
Asian/Pacific Islander	6.1%			
Black	0.7%			
Ambulatory assessment				
TST	432.68 (138.98)	0.19 [0.15; 0.24]	155.25 (79.87)	0.00–407.10
SOL	16.50 (29.31)	0.18 [0.14; 0.23]	26.95 (24.21)	0.00–130.86
SE	83.31 (8.29)	0.26 [0.21; 0.32]	8.49 (3.82)	0.00–25.83
WASO	53.21 (33.46)	0.26 [0.21; 0.32]	33.84 (18.05)	0.00–132.40
SI	1.80 (1.28)	0.50 [0.44; 0.57]	0.88 (0.67)	0.00–2.59
NSSI Acts	1.38 (0.93)	0.51 [0.45; 0.58]	0.62 (0.56)	0.00–3.17
SRI	4.40 (2.72)	0.24 [0.19; 0.29]	2.87 (0.88)	1.16–5.24

Abbreviations: CI, confidence interval; ICC, intraclass correlation; LB, lower bound; *M*, mean; NSSI, non‐suicidal self‐injury; RMSSD, root mean square of successive differences; SD, standard deviation; SE, sleep efficiency; SI, suicidal intent; SOL, sleep onset latency; SRI, sleep related impairments; TST, total sleep time; UB, upper bound; WASO, wake after sleep onset.

**TABLE 2:** Repeated measures correlations.

	1. SE	2. SOL	3. WASO	4. TST	5. Sleep1	6. Sleep2	7. SI	8. NSSI acts
1.	—							
2.	**−0.559**	—						
3.	**−0.290**	**−0.037**	—					
4.	**0.366**	**−0.096**	**0.353**	—				
5	−0.034	−0.026	−0.003	**−0.097**	—			
6.	−0.035	**−0.039**	−0.010	**−0.090**	**0.799**	—		
7.	0.013	−0.024	0.012	−0.018	**0.116**	**0.130**	—	
8.	−0.015	0.014	0.012	−0.002	**0.073**	**0.072**	**0.261**	—

*Note:* Significant parameter estimates are in bold at *p* < 0.05.

Abbreviations: NSSI, non‐suicidal self‐injury; SE, sleep efficiency; SI, suicidal intent; SOL, sleep onset latency; TST, total sleep time; WASO, wake after sleep onset.

### 4.1 Suicidal Intent

Within‐person variations in any objectively assessed sleep indices (i.e., SOL, SE, WASO, and TST) were unrelated to same‐day suicidal intent (*p*'s = 0.237–0.572; see Table 3). However, higher ratings of daily SRIs were significantly concurrently associated with higher ratings of same‐day suicidal intent (𝛽 = 0.059, 95% CI [0.044, 0.076], *p <* 0.001; see Table 3).

**TABLE 3:** Linear mixed effect models predicting same‐day suicidal intent.

Actigraphy derived objective sleep indices									
		Value	SE	df	*t*‐value	*p*	95% CI [LB; UB]	*|*00	*⌠* ^2^
TST	(Intercept)	1.767	0.082	2567	21.667	< 0.001	1.607; 1.927	0.817	0.767
	IV	−0.0001	0.0001	2567	−0.954	0.340	−0.0004; 0.0001		
SOL	(Intercept)	1.768	0.081	2567	21.663	< 0.001	1.608; 1.928	0.818	0.766
	IV	−0.0007	0.001	2567	−1.183	0.237	−0.0021; 0.0005		
SE	(Intercept)	1.768	0.081	2567	21.673	< 0.001	1.608; 1.928	0.817	0.766
	IV	0.0016	0.002	2567	688	0.492	−0.0031; 0.006		
WASO	(Intercept)	1.768	0.081	2567	21.668	< 0.001	1.576; 2.206	0.817	0.766
	IV	0.0003	0.001	2567	0.565	0.572	−0.0008; 0.0015		

Subjectively appraised sleep variables									
		Value	SE	df	*t*‐value	*p*	95% CI [LB; UB]	*|*00	*⌠* ^2^
	(Intercept)	1.782	0.082	3147	21.697	< 0.001	1.621; 1.943	0.838	0.804
	**SRI**	0.**059**	0.**008**	**3147**	**7.292**	**< 0.001**	0.**044; 0.076**		

*Note:* Significant parameter estimates are in bold.

Abbreviations: AIC, Akaike Information Criterion; BIC, Bayesian information criterion; CI, confidence interval; df, degrees of freedom; IV, independent variable; LB, lower bound; SE, sleep efficiency; SE, standard error; SOL, sleep onset latency; SRI, sleep related impairments; TST, total sleep time; UB, upper bound; WASO, wake after sleep onset.

Within the next‐day objective sleep index models, higher past‐day ratings of suicidal intent significantly predicted higher ratings of next‐day suicidal intent (𝛽 = 0.152, 95% CI [0.11, 0.192], *p*s < 0.001); however, no within‐person variations in objectively assessed sleep indices significantly predicted next‐day ratings of suicidal intent (*ps* = 0.062–0.425; see Table 4). Finally, within the SRI models, higher within‐person changes in past‐day suicidal intent significantly predicted higher next‐day suicidal intent (𝛽 = 0.169, 95% CI [0.133, 0.206], *p <* 0.001); however, within‐person changes in daily SRI did not significantly predict next‐day suicidal intent (*p* = 0.318; see Table 4).

**TABLE 4:** Linear mixed effect models predicting next‐day suicidal intent.

Actigraphy derived objective sleep indices									
		Value	SE	df	*t*‐value	*p*	95% CI [LB; UB]	*|*00	*⌠* ^2^
TST	(Intercept)	1.810	0.086	2337	21.111	< 0.001	1.642; 1.978	0.892	0.766
	**Past‐day SI**	**0.152**	0.**021**	**2337**	**7.365**	**< 0.001**	0.**111; 0.192**		
	IV	−0.0002	0.0001	2337	−1.018	0.309	−0.0004; 0.0001		
SOL	(Intercept)	1.810	0.086	2337	21.102	< 0.001	1.605; 2.2654	0.893	0.765
	**Past‐day SI**	0.**152**	0.**021**	**2337**	**7.361**	**< 0.001**	0.**111; 0.192**		
	IV	−0.0006	0.0007	2337	−0.797	0.425	−0.002; 0.0008		
SE	(Intercept)	1.811	0.086	2337	21.108	< 0.001	1.642; 1.978	0.893	0.765
	**Past‐day SI**	0.**152**	0.**021**	**2337**	**7.365**	**< 0.001**	0.**111; 0.192**		
	IV	0.003	0.003	2337	0.972	0.331	−0.003; 0.008		
WASO	(Intercept)	1.810	0.086	2337	21.111	< 0.001	1.642; 1.978	0.892	0.765
	**Past‐day SI**	0.**152**	0.**021**	**2337**	**7.397**	**< 0.001**	0.**111; 0.192**		
	IV	−0.0012	0.0006	2337	−1.871	0.062	−0.002; 0.00006		

Subjectively appraised sleep variables									
		Value	SE	df	*t*‐value	*p*	95% CI [LB; UB]	*|*00	*⌠* ^2^
	(Intercept)	1.827	0.167	2814	21.584	< 0.001	1.648; 2.301	0.893	0.799
	**Past‐day SI**	0.**169**	0.**019**	**2814**	**9.113**	**< 0.001**	0.**133; 0.206**		
	SRI	−0.008	0.009	2834	−0.999	0.318	−0.026; 0.008		

*Note:* Significant parameter estimates are in bold.

Abbreviations: AIC, Akaike Information Criterion; BIC, Bayesian Information Criterion; CI, confidence interval; df, degrees of freedom; IV, independent variable; LB, lower bound; SE, sleep efficiency; SE, standard error; SI, suicidal intent; SOL, sleep onset latency; SRI, sleep related impairments; TST, total sleep time; UB, upper bound; WASO, wake after sleep onset.

### 4.2 NSSI

No within‐person variations in any objectively assessed sleep indices (i.e., SOL, SE, WASO, and TST) were significantly related to same‐day NSSI frequency (*p*s = 0.185–0.671; see Table 5). However, higher ratings of daily SRIs were significantly concurrently associated with higher same‐day NSSI frequency (*B* = 0.143, SE = 0.026, *p <* 0.001; see Table 5).

**TABLE 5:** Cumulative link mixed effects models predicting same‐day non‐suicidal self‐injury.

Actigraphy derived objective sleep indices								
	Value	SE	z‐value	*p*	95% CI [LB; UB]	OR	logLik	*|*00
TST	−0.0002	0.0005	−0.425	0.671	−0.001; 0.001	0.999	−1453.24	3.174
SOL	0.002	0.002	1.326	0.185	−0.001; 0.006	1.002	−1452.63	3.182
SE	−0.006	0.007	−0.853	0.393	−0.021; 0.008	0.993	−1452.98	3.181
WASO	0.001	0.002	0.736	0.462	−0.002; 0.004	1.001	−1453.13	3.279

Subjectively appraised sleep variable								
	Value	SE	z‐value	*p*	95% CI [LB; UB]	OR	logLik	*|*00
**SRI**	0.**143**	0.**026**	**5.425**	**< 0.001**	0.**091; 0.194**	**1.153**	**−1762.32**	**3.249**

*Note:* Significant parameter estimates are in bold.

Abbreviations: AIC, Akaike Information Criterion; CI, confidence interval; LB, lower bound; logLik, log likelihood; OR, odd ratio; SE, sleep efficiency; SE, standard error; SOL, sleep onset latency; SRI, sleep related impairments; TST, total sleep time; UB, upper bound; WASO, wake after sleep onset.

Within all the objective sleep index models, greater past‐day frequency of NSSI acts significantly predicted next‐day frequency of NSSI (*B*'s = 0.165–0.166, SE = 0.75, *p*s = 0.026–0.027); however, no within‐person variation in any objectively assessed sleep indices significantly predicted greater next‐day frequency of NSSI (*p*'s = 0.566–0.908; see Table 6). Finally, within the SRI model, increased past‐day NSSI frequency significantly predicted greater next‐day frequency of NSSI (*B* = 0.209, SE = 0.065, *p <* 0.001). However, higher past‐day ratings of daily SRI did not significantly predict greater next‐day frequency of NSSI acts (*B* = 0.031, SE = 0.031, *p* = 0.254; see Table 6).

**TABLE 6:** Cumulative link mixed effects models predicting next‐day non‐suicidal self‐injury.

Actigraphy derived objective sleep indices								
	Value	SE	z‐value	*p*	95% CI [LB; UB]	OR	logLik	*|*00
TST	−0.00005	0.0005	−0.115	0.908	−0.001; 0.001	0.999	−1349.67	2.941
**NSSI**	0.**166**	0.**075**	**2.231**	0.**026**	0.**020; 0.312**	**1.181**		
SOL	0.001	0.002	0.439	0.660	−0.003, 0.005	1.001	−1349.58	2.942
**NSSI**	0.**166**	0.**075**	**2.223**	0.**026**	0.**019; 0.312**	**1.180**		
SE	−0.004	0.008	−0.574	0.566	−0.019; 0.011	0.996	−1349.51	2.944
**NSSI**	0.**165**	0.**075**	**2.206**	0.**027**	0.**147; 0.399**	**1.180**		
WASO	0.001	0.002	0.323	0.747	−0.003; 0.004	1.001	−1349.63	2.943
**NSSI**	0.**165**	0.**075**	**2.215**	0.**027**	0.**019; 0.311**	**1.180**		

Subjectively appraised sleep variables								
	Value	SE	z‐value	*p*	95% CI [LB; UB]	OR	logLik	*|*00
SRI	0.031	0.031	1.141	0.254	−0.022; 0.085	1.032	−1622.68	3.033
**NSSI**	0.**209**	0.**065**	**3.208**	0.**001**	0.**081; 0.337**	**1.233**		

*Note:* Significant parameter estimates are in bold.

Abbreviations: AIC, Akaike Information Criterion; CI, confidence interval; LB, lower bound; logLik, log likelihood; OR, odd ratio; SE, sleep efficiency; SE, standard error; SOL, sleep onset latency; SRI, sleep related impairments; TST, total sleep time; UB, upper bound; WASO, wake after sleep onset.

### 4.3 Post Hoc Exploratory Analyses

None of the objectively assessed sleep indices demonstrated direct concurrent or prospective relationships with SI or NSSI. However, these objectively assessed indices may be driving worsening SRI appraisals over time. This assumption is substantiated by findings from Hanish et al. (2017) and Mournet et al. (2025) whereby higher TST demonstrated a significant negative relationship with PROMIS‐SRI items and self‐rated ambulatory fatigue was related to and predictive of self‐injury urges, SI, and desire to be alive, respectively. Accordingly, random intercept linear fixed effects models estimated via ML were used to test the effect of actigraphy derived objective sleep indices on within‐day (*T*) and next‐day (*T* + 1) SRIs over the course of the study follow‐up period. Each of the within‐person mean centered objective sleep indices (i.e., TST, SOL, SE, and WASO) predicting same‐day SRIs were entered into separate regression models to avoid multicollinearity. The second set of models examined the effect of past‐evening objectively assessed sleep indices on next‐day SRIs (*T* + 1). As with same‐day models, within‐person mean centered objective sleep indices and past‐day SRIs were entered into separate models, predicting next‐day SRIs for each objective index.

Results of the same‐day models suggested that lower within‐person TST (𝛽 = −0.002, 95% CI [−0.0022, −0.0001], *p <* 0.001) and SE (𝛽= −0.016, 95% CI [−0.028, −0.005], *p <* 0.001) were significantly related to greater same‐day SRI appraisals; none of the other actigraphy‐derived objective sleep indices were significantly related to same‐day SRI appraisals (*p*s > 0.05; see Table S2). Results of the next‐day models suggested that higher SRI appraisals significantly predicted higher *next‐day* SRI appraisals within all models (*p*s = < 0.001); additionally, lower within‐person past‐day TST (𝛽 = −0.001, 95% CI [−0.002, −0.0003], *p* = 0.005) significantly predicted higher next‐day SRI appraisals (see Table S3). None of the other actigraphy‐derived objective sleep indices significantly predicted next‐day SRI appraisals (*p*s > 0.05; see Table S2).

## 5 Discussion

This study examined the relationships between SRIs and objectively measured sleep quality with suicidal intent and NSSI among college students using daily diary and actigraphy‐based methodology. Hypotheses one and two were supported: objective measures of sleep quality were unrelated to same‐day and next‐day suicidal intent and NSSI frequency. Hypotheses 3 and 4 were only partially supported: SRIs were related to *same‐day*, but not next‐day, suicidal intent and NSSI frequency.

The findings align with prior research suggesting self‐reported sleep‐related impairments are more robust predictors of SI and NSSI relative to objective measurements (Glenn et al. 2021; Kivelä et al. 2023). Consistent with previous studies (Becker et al. 2018; Liu et al. 2020), participants who reported greater impairments due to sleep exhibited higher levels of same‐day, but not next‐day, suicidal intent. By contrast, none of the objectively measured sleep quality indices demonstrated direct associations with suicidal intent. Although prior research suggests that objectively measured sleep quality variability disturbance may be linked to suicide risk (Bernert et al. 2019; Littlewood et al. 2019), this study did not observe significant associations between actigraphy‐derived sleep quality indices and daily fluctuations in SI.

Stronger daily SRI may reflect broader cognitive and emotional vulnerabilities. For instance, Littlewood et al. (2017) observed negative cognitive appraisals and other psychological factors, like increased isolation due to fatigue, partially accounted for the relationship between sleep problems and SI. Others have observed that rumination, fatigue, and negative affective reactivity may explain the relationship between sleep disturbances and STB (Hamilton et al. 2023; Holdaway et al. 2018; Mournet et al. 2025). Taken together, SRIs may exacerbate individuals' experiences of hopelessness or perceptions that their distress is inescapable, driving worsening suicidal intent over days and weeks.

Indeed, subjectively appraised SRI may be more closely tied to perceived emotional distress and dysregulation, core components of the diathesis‐stress model of suicide risk (O'Connor 2011). In a qualitative investigation of the impact of sleep problems on suicide, one participant described the impact of poor sleep on distress and dysregulation: “It's really difficult … with the sort of lacking resources mentally, lacking the energy … so yeah it's very difficult after a poor night's sleep to stay positive” (Littlewood et al. 2016, 5). Negative cognitive appraisals like hopelessness, entrapment, and perceived distress are evidence‐based drivers of STB (Klonsky et al. 2018) and may explain why SRIs appear to covary with SI on a day‐to‐day basis relative to objectively measured sleep disruptions.

The findings of this study extend prior research by examining the relationship between sleep disturbances and NSSI; in fact, this study is among the first to link SRIs with NSSI *behavior* using ambulatory methods (Hysing et al. 2015; Tubbs et al. 2023). The results suggest SRI appraisals significantly predicted same‐day NSSI frequency. Certain cognitive vulnerabilities may explain why subjective and not objectively measured sleep disturbances were related to NSSI frequency. Khazaie et al. (2021) observed that emotion regulation deficits and greater depression symptom severity explained why adults with sleep problems were at higher risk of NSSI. Likewise, Wang and Sun (2024) found that rumination fully accounted for the relationship between insomnia symptoms and NSSI in a sample of college students. As such, cognitive appraisals of physiological distress due to sleep disturbances appear to be driving changes in STB rather than the objectively measured physiological processes themselves.

So far, extant research has yet to observe significant relationships between objectively measured sleep index changes and STB in ambulatory research (Glenn et al. 2021; Kivelä et al. 2023). Nevertheless, exploratory post hoc analyses suggest lower TST and lower SE were significantly related to higher same‐day SRI appraisals. Lower previous day TST also significantly predicted increases in next‐day SRI. In turn, worsening SRI appraisals appear to drive some change in suicidal intent and NSSI behavior. Such observations suggest a potential indirect relationship whereby physiological disturbances in sleep quality are driving worsening STB through SRI or other cognitive or affective impairments like negative affect reactivity and fatigue (Hamilton et al. 2023; Mournet et al. 2025). However, this indirect pathway was not fully modeled and will require future investigation.

### 5.1 Clinical Implications and Future Directions

The results suggest that self‐reported SRI may be more informative clinical indicators of STB risk than objectively appraised sleep disturbances and are a more feasible means of screening and assessment. Clinicians should consider prioritizing assessment of sleep‐related complaints when assessing suicide risk and consider implementing real‐time self‐monitoring tools to track fluctuations in SRI and SI during treatment. For clinicians working primarily with patients with sleep‐related concerns, follow‐up questions concerning the impact of poor sleep on daily functioning and SI and NSSI as concurrent concerns are clinically indicated. This may be particularly applicable for primary care physicians and outpatient behavioral health providers attempting to identify suicide risk via indirect means. Screening for SRI using the PROMIS‐SF may facilitate helpful and clinically indicated conversations about STB. Considering SRIs were significantly associated with suicidal intent and NSSI over time, interventions targeting both objective sleep disruptions *and* the negative cognitive appraisals of sleep quality may be particularly useful suicide prevention initiatives.

Cognitive‐Behavioral Therapy for Insomnia (CBT‐i) could be integrated into suicide risk treatment protocols for college students given some patients report improvements in SI following the completion of the CBT‐i protocol (Jernelöv et al. 2021). Other evidence‐based treatments for STB that integrate and “front load” elements of CBT‐i (e.g., stimulus control) are highly effective and may yield more rapid treatment gains (Bryan et al. 2025). These and other interventions (e.g., Acceptance and Commitment Therapy [ACT]) may be useful for undermining negative cognitive appraisals driving SI and NSSI frequency. Future intervention research may be able to determine the degree to which improvements in SRI and objective metrics of sleep quality are in turn related to improvements in SI and NSSI frequency.

The current results, in the context of others previously published (Czyz et al. 2023; Glenn et al. 2021; Kivelä et al. 2023), also raise questions about the *current* utility of passive sensing technology and actigraphy, specifically to assess concurrent and short‐term risk for STB. Incorporating multi‐method assessment approaches that combine subjective and objective sleep measures may be optimal and enhance understanding of how sleep disturbances (and other physiological processes) contribute to risk for suicidal behavior.

### 5.2 Limitations

This study should be interpreted within the context of several limitations. The study relied on a college student sample, which may limit the generalizability of the findings to other populations. Although a strength in the context of risk detection, the high proportion of participants in counseling or taking medication also limits the generalizability of the current findings. Next, while the weekly laboratory visits and safety checks were ethically necessary, these procedures likely impacted participants' natural sleep and self‐reported STB patterns due to self‐monitoring effects (see Knorr and Ammerman 2026). Follow‐up research examining long‐term and individual‐level change patterns in sleep disturbances and suicide risk is also needed; the use of random intercept models limits insights into individual differences in sleep patterns over time. Future research should prioritize studies designed to support random slopes models. Additionally, the actigraphy‐derived sleep indices used may not have captured all relevant sleep disturbances and adherence issues with passive sensor data collection, particularly among individuals experiencing more severe SI, which may contribute to null findings (Jiang et al. 2023). Finally, the observed effects of SRIs on suicidal intent and NSSI, though significant, were small, suggesting additional factors may contribute to these outcomes.

## 6 Conclusions

This study provides important insights into the role of SRIs in suicide risk among high risk college students. SRIs were significantly associated with same‐day suicidal intent and NSSI, underscoring the importance of subjectively reported cognitive and affective impairments due to sleep in suicide prevention. Interventions targeting sleep disturbances and related impairments may be particularly effective in mitigating suicide risk (e.g., Brief Cognitive Behavioral Therapy [BCBT]; Bryan et al. 2025). Future research should continue to explore the mechanisms underlying the relationship between SRIs and suicidal behaviors and investigate novel methodologies for improving sleep disturbance assessments in suicide risk populations.

## Author Contributions

**Nicolas Oakey‐Frost:** conceptualization, writing – original draft, writing – review and editing, formal analysis, methodology. **Megan L. Rogers:** methodology, validation, writing – review and editing, supervision. **Jennifer J. Muehlenkamp:** investigation, funding acquisition, methodology, writing – original draft, project administration, resources, data curation, supervision. **Emma H. Moscardini:** wrtiting – original draft, writing – review and editing.

## Funding

Research reported in this publication was supported by the National Institute of Mental Health 2R15MH110960. The content is solely the responsibility of the authors and does not necessarily represent the official views of the National Institutes of Health.

## Ethics Statement

This study was reviewed and approved by the University of Wisconsin‐Eau Claire on March 29th, 2022 (IRB #MUEHJJ183732020). All participants included in the analyzed data set provided informed consent.

## Conflicts of Interest

The authors declare no conflicts of interest.

## Supporting information


**Tables S1–S3:** sltb70091‐sup‐0001‐TableS1‐S3.docx.

## Data Availability

The data that support the findings of this study are available on request from the corresponding author. The data are not publicly available due to privacy or ethical restrictions.
