# Supplementary material for: Comparing Objective Measures of Sleep Disturbance and Sleep Related Impairments as Proximal Risk Indicators of Suicidal Intent and Non‐Suicidal Self‐Injury
Source: Suicide Life Threat Behav. 2026 Mar 31;56(2):e70091. doi: 10.1111/sltb.70091 (PMC13036479; doi:10.1111/sltb.70091)
Supplement: Supplementary file 1 — Tables S1–S3: sltb70091‐sup‐0001‐TableS1‐S3.docx. [file SLTB-56-0-s001.docx]

| **Supplemental Table 1**  Daily Diary Item Level Missingness | | |
| --- | --- | --- |
| **Variable** | **Number Missing** | **% Missing** |
| SOL | 685 | 18.4% |
| SE | 685 | 18.4% |
| WASO | 685 | 18.4% |
| TST | 685 | 18.4% |
| Sleep 1 | 444 | 11.9% |
| Sleep 2 | 449 | 12.1% |
| Suicidal Intent | 443 | 11.9% |
| NSSI Acts | 441 | 11.8% |
| Note. SOL=Sleep Onset Latency; SE=Sleep Efficiency; WASO=Wake After Sleep Onset; TST=Total Sleep Time; NSSI=Non-suicidal self-injury | | |

| **Supplemental Table 2** | | | | | | | | | |
| --- | --- | --- | --- | --- | --- | --- | --- | --- | --- |
| Linear mixed effects models predicting **same-day** sleep related impairments (SRI) | | | | | | | | | |
| *Actigraphy Derived Objective Sleep Indices* | | | | | | | | | |
|  |  | *Value* | *SE* | *df* | *t-value* | *p-value* | *95% CI [LB; UB]* | *τ00* | *σ^2^* |
| TST | (Intercept) | 4.437 | .126 | 2907 | 35.294 | < .001 | 4.191; 4.684 | 1.792 | 5.476 |
|  | **IV** | **-.002** | **.0003** | **2907** | **-4.574** | **< .001** | **-.0022; -.0001** |  |  |
|  |  |  |  |  |  |  |  |  |  |
| SOL | (Intercept) | 4.437 | .126 | 2907 | 35.295 | < .001 | 4.191; 4.683 | 1.791 | 5.512 |
|  | IV | -.0023 | .0016 | 2907 | -1.412 | .158 | -.006; .0001 |  |  |
|  |  |  |  |  |  |  |  |  |  |
| SE | (Intercept) | 4.437 | 0.126 | 2907 | 35.294 | < .001 | 4.191; 4.684 | 1.792 | 5.501 |
|  | **IV** | **-.016** | **.006** | **2564** | **-2.754** | **.006** | **-.028; -.005** |  |  |
|  |  |  |  |  |  |  |  |  |  |
| WASO | (Intercept) | 4.437 | .126 | 2907 | 35.295 | < .001 | 4.191; 4.684 | 1.791 | 5.515 |
|  | IV | .0001 | .002 | 2907 | .131 | .896 | -.0027; .0032 |  |  |
| Note. TST=total sleep time; SOL=sleep onset latency; SE=sleep efficiency; WASO=wake after sleep onset; IV=independent variable; SI=suicidal intent; SE=standard error; df=degrees of freedom; CI=confidence interval; LB=lower bound; UB=upper bound; AIC=Akaike Information Criterion; BIC=Bayesian Information Criterion. **Significant parameter estimates are in bold.** | | | | | | | | | |

| **Supplemental Table 3** | | | | | | | | | |
| --- | --- | --- | --- | --- | --- | --- | --- | --- | --- |
| Linear mixed effects models predicting **next-day** sleep related impairments (SRI) | | | | | | | | | |
| *Actigraphy Derived Objective Sleep Indices* | | | | | | | | | |
|  |  | *Value* | *SE* | *df* | *t-value* | *p-value* | *95% CI [LB; UB]* | *τ00* | *σ^2^* |
| TST | (Intercept) | 5.023 | .123 | 2349 | 40.764 | < .001 | 4.782; 5.264 | 1.309 | 1.913 |
|  | **Past-day SRI** | **.155** | **.020** | 2349 | **7.612** | **< .001** | **.115; .196** |  |  |
|  | **IV** | **-.001** | **.0003** | **2349** | **-2.837** | **.005** | **-.002; -.0003** |  |  |
|  |  |  |  |  |  |  |  |  |  |
| SOL | (Intercept) | 5.024 | .123 | 2349 | 40.713 | < .001 | 4.782; 5.266 | 1.311 | 1.916 |
|  | **Past-day SRI** | **.161** | **.020** | 2349 | **7.907** | **< .001** | **.121; .201** |  |  |
|  | IV | .0002 | .002 | 2349 | .169 | .866 | -.003 .003 |  |  |
|  |  |  |  |  |  |  |  |  |  |
| SE | (Intercept) | 5.023 | .123 | 2349 | 40.721 | < .001 | 4.782; 5.265 | 1.311 | 1.914 |
|  | **Past-day SRI** | **.159** | **.020** | 2349 | **7.831** | **< .001** | **.119; .199** |  |  |
|  | IV | -.010 | .006 | 2349 | -1.856 | .064 | -.021; .001 |  |  |
|  |  |  |  |  |  |  |  |  |  |
| WASO | (Intercept) | 5.024 | .123 | 2349 | 40.731 | < .001 | 4.782; 5.266 | 1.310 | 1.915 |
|  | **Past-day SRI** | **.161** | **.020** | 2349 | **7.898** | **< .001** | **.121; .201** |  |  |
|  | IV | -.002 | .001 | 2349 | -1.421 | .155 | -.005; .0007 |  |  |
| Note. TST=total sleep time; SOL=sleep onset latency; SE=sleep efficiency; WASO=wake after sleep onset; IV=independent variable; SI=suicidal intent; SE=standard error; df=degrees of freedom; CI=confidence interval; LB=lower bound; UB=upper bound; AIC=Akaike Information Criterion; BIC=Bayesian Information Criterion. SRI=sleep related impairments. **Significant parameter estimates are in bold.** | | | | | | | | | |
